# Time course of muscle activation, energetics and mechanics of running in minimalist and traditional cushioned shoes during level running

**DOI:** 10.1038/s41598-023-31984-z

**Published:** 2023-03-27

**Authors:** Gilles Udin, Aitor Fernandez Menendez, Jonas Hoyois, Mathias Chevalier, Davide Malatesta

**Affiliations:** Institute of Sport Sciences of University of Lausanne (ISSUL), University of Lausanne, Bâtiment Synathlon, 1015 Lausanne, Switzerland

**Keywords:** Physiology, Metabolism

## Abstract

The study aimed to compare the ankle muscles activation, biomechanics and energetics of running in male runners during submaximal level run using minimalist (MinRS) and traditional cushioned (TrdRS) running shoes. During 45-min running in MinRS and TrdRS, the ankle muscles pre- and co-activation, biomechanics, and energetics of running of 16 male endurance runners (25.5 ± 3.5 yr) were assessed using surface electromyography (tibialis anterior and gastrocnemius lateralis), instrumented treadmill and indirect calorimetry, respectively. The net energy cost of running (C_r_) was similar for both conditions (*P* = 0.25) with a significant increase over time (*P* < 0.0001). Step frequency (*P* < 0.001), and total mechanical work (*P* = 0.001) were significantly higher in MinRS than in TrdRS with no evolution over time (*P* = 0.28 and *P* = 0.85, respectively). The ankle muscles pre- and co-activation during the contact phase did not differ between the two shoe conditions (*P* ≥ 0.33) or over time (*P* ≥ 0.15). In conclusion, during 45-min running, Cr and muscle pre- and co-activation were not significantly different between MinRS and TrdRS with significantly higher step frequency and total mechanical work noted in the former than in the latter. Moreover, C_r_ significantly increased during the 45-min trial in both shoe conditions along with no significant change over time in muscle activation and biomechanical variables.

## Introduction

Minimal running shoes (MinRS) have gained popularity over the last two decades. Among their supposed advantages, MinRS have been advocated to improve the running economy—the energy cost of running (C_r_; the energy expenditure per unit of distance)—and reduce the impact peak and loading rate by promoting a forefoot strike^1^. Running economy is a key determinant of aerobic long-distance performance^2^. Although running biomechanics occurring during the propulsion phase seem to have the strongest direct link with running economy, it is difficult to identify the main biomechanical factors involved in improving running economy in MinRS, and inconsistent evidence still exists^3^. Therefore, it seems pivotal to assess running economy along with biomechanical running parameters and total mechanical work to evaluate mechanical efficiency (i.e., the mechanical work divided by C_r_). In fact, the latter concomitantly takes into account the energetics and mechanics of running and may provide valuable insight into running patterns, particularly into mechanical energy saving mechanisms.

Despite the expected benefits, the actual MinRS advantages on running economy are difficult to establish^4^, particularly when focusing on the transitioning phase to MinRS^5^. However, a modest but significant running economy advantage in MinRS compared with traditional cushioned running shoes (TrdRS) was reported regardless of the strike type^6,7^, which was confirmed by meta-analyses^6^. Although this slight running economy advantage in MinRS may be associated to their lower mass^8^ compared with TrdRS, the contributing biomechanical factors involved in this higher running economy seems difficult to demonstrate. In fact, MinRS may alter the biomechanics of running compared with TrdRS inducing forefoot strike^9^, shorter ground contact time (t_c_)^10^, increased step frequency^11^ and leg stiffness (k_leg_; the ratio between maximal vertical ground reaction force and lower limb deformation, which characterizes the elastic property of the linear lower limb spring)^12^. This increased k_leg_ associated with forefoot strike may result in a higher storage-release of elastic energy in the muscle–tendon unit during running [the stretch–shortening cycle^13^] in MinRS than in TrdRS. An optimal k_leg_ is a tradeoff involving several constraints; a stiffer lower limb may imply shorter t_c_ and lower vertical displacements of the center of mass, both of which may be involved in decreasing C_r_^14^ and increasing running mechanical efficiency^15^. On the other hand, increasing K_leg_ above this optimal value during running may induce higher loads related to increasing risks of injury and increased muscle activation associated with an increased metabolic cost^16^.

Agonist ankle muscle preactivation (muscle activation prior to ground contact) and ankle muscle coactivation (simultaneous activation of agonist and antagonist muscles during ground contact) were both increased in forefoot strike pattern^17^ and may contribute to increased storage-release of elastic energy^18,19^ and k_leg_^20^ during running in MinRS but at the expense of C_r_. These findings suggest that an optimal interplay may exist between lower limb muscle preactivation and coactivation, k_leg_ and running economy and that this equilibrium might be changed in MinRS and may penalize runners with running duration in long-running sessions, especially during the phase of transitioning to MinRS^7,21^. However, the biomechanical and energy running comparison between MinRS and TrdRS is often restrained to a short bout of running (5–10 min). Only two studies investigated the effect of running duration in MinRS vs. TrdRS^22,23^, demonstrating a specific increase in running economy exclusively with MinRS^23^ associated with distinct biomechanical changes^22,23^ in two footwear conditions over time (a more pronounced decrease in k_leg_ and increase in footstrike angle in MinRS than in TrdRS). However, one study examined the effect of footwear on the running economy and biomechanics only after a short-distance trail^23^. This design did not allow the authors to compare energetic and biomechanical changes during running in two footwear conditions (i.e., time course of changes)^23^. Another study^22^ only investigated the biomechanical changes during 50 min of running composed of 5 blocks of 5 min of flat, ascent (5%) and descent (-5%) treadmill running. This mixed profile of the trial used in this study^22^ (different slope conditions) may have influenced the comparison during running between the two footwear conditions.

Therefore, the aim of this study was to compare the ankle muscle pre- and co-activation and biomechanics and energetics of running in healthy male endurance runners during 45 min of level ground running (sampled at 5, 15, 30 and 45 min) at 95% of the ventilatory threshold using MinRs and TrdRS. We hypothesized that (1) at the beginning of the trial (5 min), C_r_ would be lower and step frequency, k_leg_ and ankle muscle preactivation and coactivation would be higher in MinRS compared with TrdRS; and (2) a more pronounced increase in C_r_ and ankle muscle activations associated with a greater decrease in k_leg_ would be found over exercise duration in MinRS compared with TrdRS. Finally, we also explored the changes in the total mechanical work and efficiency in MinRs vs TrdRS during 45-min running.

## Methods

### Participants

Sixteen healthy male experienced endurance runners (25.5 ± 3.5 yr; 1.85 ± 0.06 m; 73.6 ± 5.7 kg; personal best record for running 10 km: 39.6 ± 1.8 min) volunteered and provided written informed consent to participate in this study. All participants were regular rearfoot runners using TrdRS with no major injuries in the past 3 months, no lower extremity abnormalities, and a reference time of 10 km of 40 min or less.

### Experimental design

Participants visited the laboratory on three occasions. In the first experimental session, participants’ anthropometric assessments and personal and training information were collected. Then, using their usual TrdRS, the participants performed a submaximal incremental running test until the respiratory exchange ratio (RER) reached 1.0 on an instrumented treadmill (T150–FMT-MED, Arsalis, Belgium) to assess their first ventilatory threshold. At the end of this first visit, a pair of FiveFingers (Vibram, Italy) was given to each runner as MinRS to perform a familiarization protocol period of 2 weeks involving a gradual exposition to minimalistic shoes over 4 training sessions (first session: 5 min; second session: 10 min; third session: 15 min; fourth session: 20 min) performed at moderate running intensity similar to that used in the last two visits. This familiarization must be performed before the last 2 experimental sessions, in which each runner performed a continuous submaximal running test for 45 min at 95% of the first ventilatory threshold once using TrdRS and once again using MinRS in randomized order. For the 2 experimental conditions, the mass of the shoes was paired (320 g) using small lead strips firmly attached to the uppers of the running shoes^8^. The metabolic data were continuously collected in each experimental session, whereas the biomechanical data were assessed in the second and third sessions at 5, 15, 30 and 45 min of the continuous submaximal running test.

### Exercise testing

#### Submaximal incremental running test

After a 5-min rest period on the treadmill, participants ran for a 10-min warm-up at a speed of 8 km h^−1^, which was followed by an increase of 1 km h^−1^ every 3 min until the RER reached 1.0. During the tests, oxygen uptake and CO_2_ output were measured continuously using a breath-by-breath online system (Oxycon Pro, Jaeger, Würzburg, Germany). Before each test, the metabolic card was calibrated with 16% O_2_ and 5% CO_2_ at low, medium and high flow rates utilizing a 3-l air syringe according to the manufacturer’s recommendations. The first ventilatory threshold was determined as described in the literature using Wasserman’s ventilatory method^24^, which consists of visually determining the point at which the ventilatory equivalent for O_2_ increases while the ventilatory equivalent for CO_2_ remains stable. To support this estimate of the first ventilatory threshold, we then used the Beaver ventilatory method^24^ determining the inflection point of the CO_2_ output with respect to the oxygen uptake. Three blinded and independent investigators determined the first ventilatory threshold.

#### Submaximal continuous running test

After a 5-min rest period and a standardized 10-min warm-up at 10 km h^−1^, the runners performed a 45-min submaximal continuous running exercise at an intensity corresponding to 95% of the first ventilatory threshold determined during the submaximal incremental test. Gas exchanges were continuously measured and used to assess the ventilation, RER and net energy cost of running at 5, 15, 30 and 45 min during the test. At these time points, heart rate and biomechanical data (20 consecutive steps) were also assessed using an heart rate monitor (S810i, Polar Electro OY, Finland) and an instrumented treadmill, respectively (please see paragraph “Assessments” for details).

### Assessments

#### Net energy cost of running

Breath-by-breath oxygen uptake data were initially examined to exclude errant breaths due to coughing or swallowing [oxygen uptake values ≥ 3 standard deviations (SD) from the local mean were deleted]. Then, the oxygen uptake, ventilation and RER values from the last minute before the 5th, 15th, 30th and 45th minutes were averaged. The oxygen uptake values were then divided by body mass and converted to gross metabolic rates using a standard equation^25^ to calculate the energy equivalent of 1 L of oxygen (Eq.O_2_; Eq. 1).

1 $$Eq. {O}_{2}= 21.13\left[\frac{(RER-0.7)}{0.3}\right]+ 19.6\left[\frac{(1-RER)}{0.3}\right]$$

The metabolic rate during standing was subtracted from all gross metabolic rates to calculate the net metabolic rate at each time point. Then, these latter values were divided by the running speed to obtain the net energy cost of running (C_r_, J kg^−1^ m^−1^).

#### Biomechanics of running

##### Spatiotemporal parameters

For each experimental condition at 5, 15, 30 and 45 min, t_c_, step frequency and length were assessed during 20 consecutive steps using the instrumented treadmill and acquired at a sample rate of 1000 Hz. For 30 s of each time point, the ground reaction forces were assessed in the vertical, horizontal and lateral components. Foot contact and toe-off events were determined based on the 20-N vertical force threshold level. The contact time was defined as the period during which the vertical force was greater than 20 N. The step length and frequency were assessed as the distance and the inverse of the duration between two consecutive foot contacts, respectively.

##### Mechanical works

The external mechanical work of running (J kg^−1^ m^−1^), which is defined as the mechanical work performed to move the center of mass, was computed as the sum between the fore-aft and lateral and vertical mechanical works^15,26^. These mechanical works were obtained from 3D forces allowing the computation of the velocity in all three directions (V_v_, V_h_, and V_l_) and vertical displacement of the center of mass over time by single mathematical integration of the three accelerations (obtained by the ground reaction force measurements) and by double mathematical integration of the vertical acceleration, respectively^26^. The fore-aft and lateral and vertical mechanical works were calculated as the sum of positive increments in the fore-aft and lateral kinetic (Eq. 2) and vertical (Eq. 3) energies of the center of mass over a complete step, respectively.

2 $${E}_{kfl}={E}_{kf}+{E}_{kl}=0.5m\left({V}_{h}^{2}\right)+0.5m\left({V}_{l}^{2}\right)$$

where *m* is the body mass.

3 $${E}_{v}={E}_{p}+{E}_{kv}=mg{s}_{v}+ 0.5m\left({V}_{v}^{2}\right)$$

where *m* is the body mass, *s*_*v*_ is the vertical displacement of the center of body mass and *g* is the acceleration of gravity (g = 9.81 m s^−2^).

The mechanical internal work (W_int_ in J kg^−1^ m^−1^), which is the work performed to move the limbs around the center of mass, was estimated using the formula of Nardello et al.^27^ (Eq. 4).

4 $${W}_{int}=SF\cdot v\cdot \left(1+{\left(\frac{DF}{1-DF}\right)}^{2}\right)\cdot 0.08$$

where SF is the stride frequency, *v* is the running speed, DF is the duty factor [the fraction of the duration of the stride period when each foot is on the ground^27^] and 0.08 is a compound dimensionless term accounting for the inertial properties of the limbs and the mass partitioned between the limbs and the rest of the body^27^.

Total positive mechanical work performed per distance traveled was evaluated as the sum of the external mechanical work and internal mechanical work^28^.

##### Leg stiffness

The leg stiffness (k_leg_ in N m^−1^) was calculated as a ratio of the maximal vertical force (F_v,max_ in N) during contact to the lower limb deformation (i.e., the peak displacement of the leg spring in m) (∆L in m; Eq. 5).

5 $${k}_{leg}=\frac{{F}_{v,max}}{\Delta L}$$

where ∆L is calculated from Eq. 6 according to Morin et al.^29^.

6 $$\Delta L=L-\sqrt{{{L}^{2}-\left(\frac{v\cdot {t}_{f}}{2}\right)}^{2}}+\Delta y$$

where L is the initial leg length (great trochanter-to-ground distance in a standing position) (m), v is the running speed (m s^−1^), t_f_ is the fly time (s) and ∆y is the downward displacement of the center of mass.

##### Braking and propulsive durations and forces and loading, braking and propulsive rates

Push and brake time and their ratio were assessed. The peaks of braking and propulsive forces were determined as the minimum and maximum of the horizontal force during the contact phase, respectively. The braking and propulsive rates were calculated as the slope of the horizontal force between 20 and 80% of the period between the initial contact and minimum of the horizontal force and between the mid-stance time and maximum of the horizontal force, respectively. The loading rate was computed as the slope of the vertical force between 20 and 80% of time between initial contact and 15% of the stance phase^30,31^. This method was chosen to have a uniform measurement across runners and conditions. All these values were then normalized by body mass.

### Mechanical efficiency

The mechanical efficiency was defined as the ratio of the total positive mechanical work to C_r_^28^ both expressed in J kg^−1^ m^−1^.

### Muscle activation and coactivation

Surface electromyography (EMG) was obtained from the tibialis anterior (TA) and gastrocnemius lateralis (GL) during both shoe condition trials (EMG100C, Biopac Systems Inc., Hilliston, MA, USA). Prior to placement, the skin areas were prepared (shaved, abraded and cleaned with alcohol to lower skin impedance), and two surface electrodes (Ag/AgCl, 11 mm diameter) were placed on the muscle location according to SENIAM guidelines^32^. The signals were treated using dedicated software (Biopac Systems Inc., Hilliston, MA, USA). The raw digital EMG signals were filtered (20 Hz – 450 Hz), fully rectified and low-pass filtered (20 Hz). Preactivation was defined as the muscle activity during the 100 ms before the foot contact (preactivation phase) normalized by the muscle activity obtained during the warm-up at 10 km/h. The preactivation ratio was calculated by dividing the normalized TA preactivation by the normalized GL preactivation (TA/GL preactivation ratio). Muscle activation was defined as a deviation of 5 standard deviations from the resting EMG baseline^33^. Coactivation of GL and TA was determined as the time when both muscles were simultaneously active during the stance phase and expressed as a percentage of total stride duration^33^.

### Rating of perceived effort and postexercise lower limb muscle pain

Participants were asked to rate their perceived exertion during running at 5, 15, 30 and 45 min using the Borg rating of perceived exhaustion scale^34^. Postexercise muscle pain of the lower limbs was assessed at 24 h, 48 h and 72 h using a 0–10 visual analog scale.

### Statistical analysis

All statistical analyses were performed with SPSS version 24 (SPSS, Chicago, IL). Data are expressed as means ± SD for all variables. A two-way repeated measures ANOVA [time (5, 15, 30 and 45 min) × shoe condition (MinRS vs, TrdRS)] was used to determine differences in muscle coactivation, energetics and biomechanics of running. When the assumption of sphericity was violated, Green-House-Geisser or Huynh–Feldt adjustments were employed where appropriate. When repeated-measures ANOVA revealed a significant main effect (time or shoes condition) or interaction effect, multiple comparisons with Bonferroni adjustments were performed to test the significance of the differences. To minimize the type 1 errors due to the 30 ANOVAs used to determine differences in the experimental variables of this study, the level of significance was set as *P* ≤ 0.0016 (i.e., 0.05/30).

### Ethics approval and consent to participate

This study was approved by the local ethics committee (Cantonal Swiss Ethics Committees on research involving humans; CER-VD 468/13) and all the participants provided written informed consent. All the participants provided written informed consent for participation and publication.

## Results

The F, P and partial eta squared values for the two-way repeated measures ANOVAs used in this study are reported in Table 1.

**Table 1:** F, P and partial eta squared values for the two-way repeated measures ANOVAs [time (5, 15, 30 and 45 min) × shoe condition (MinRS vs, TrdRS)].

Variable	Condition			Time			Interaction		
	F	P	Partial eta squared	F	*P*	Partial eta squared	F	P	Partial eta squared
C_r_	F_1,15_ = 1.45	0.25	0.09	F_3,45_ = 29.80	< 0.0001	0.67	F_3,45_ = 0.33	0.81	0.02
Ventilation	F_1,15_ = 0.35	0.56	0.02	F_1.8,27.2_ = 21.04	< 0.0001	0.58	F_3,45_ = 0.26	0.85	0.79
RER	F_1,15_ = 0.67	0.44	0.04	F_2.24, 33.6_ = 34.30	< 0.0001	0.70	F_3,45_ = 0.09	0.97	0.01
Heart rate	F_1,15_ = 2.56	0.13	0.15	F_1.34,20.02_ = 82.89	< 0.0001	0.85	F_3,45_ = 0.86	0.47	0.05
Contact time (t_c_)	F_1,15_ = 5.56	0.03	0.27	F_3,45_ = 1,62	0.20	0.10	F_3,45_ = 1.43	0.25	0.09
Step Length	F_1,15_ = 15.49	0.001	0.51	F_3,45_ = 1.75	0.20	0.11	F_3,45_ = 1.25	0.32	0.08
Step frequency	F_1,15_ = 17.34	< 0.001	0.54	F_3,45_ = 1.32	0.28	0.08	F_3,45_ = 1.46	0.24	0.09
External work	F_1,15_ = 6.97	0.02	0.32	F_3,45_ = 1.11	0.35	0.07	F_3,45_ = 1.35	0.27	0.08
Vertical work	F_1,15_ = 8.69	0.01	0.37	F_3,45_ = 0.90	0.45	0.06	F_3,45_ = 1.28	0.29	0.08
Fore-aft and lateral work	F_1,15_ = 32.21	< 0.001	0.68	F_3,45_ = 1.61	0.2	0.10	F_3,45_ = 0.89	0.47	0.06
Internal work	F_1,15_ = 14.40	0.001	0.49	F_1.99,29.9_ = 0.16	0.85	0.14	F_3,45_ = 0.84	0.26	0.09
Total work	F_1,15_ = 18.54	0.001	0.55	F_3,45_ = 0.16	0.85	0.011	F_3,45_ = 1.39	0.26	0.09
F_v,max_/BM	F_1,15_ = 5.040	0.04	0.25	F_3,45_ = 0.70	0.56	0.05	F_1.82,27.26_ = 0.83	0.44	0.05
Lower limb deformation	F_1,15_ = 6.96	0.02	0.32	F_1.68,25.2_ = 0.53	0.57	0.03	F_1.26,18.87_ = 1.93	0.18	0.11
Leg stiffness (k_leg_)	F_1,15_ = 4.51	0.05	0.23	F_3,45_ = 0.16	0.92	0.011	F_3,45_ = 2.1	0.11	0.12
Push time	F_1,15_ = 1.72	0.21	0.10	F_3,45_ = 1.09	0.33	0.068	F_3,45_ = 1.04	0.33	0.07
Brake time	F_1,15_ = 17.87	< 0.001	0.54	F_3,45_ = 1.29	0.29	0.08	F_3,45_ = 0.93	0.43	0.06
Push-to-brake time ratio	F_1,15_ = 16.27	0.001	0.52	F_3,45_ = 0.28	0.69	0.018	F_3,45_ = 0.41	0.59	0.03
Loading rate	F_1,15_ = 0.25	0.63	0.02	F_3,45_ = 0.40	0.56	0.03	F_3,45_ = 1.07	0.34	0.07
Peak braking force	F_1,15_ = 0.18	0.68	0.01	F_3,45_ = 0.42	0.63	0.03	F_2.68,40.19_ = 3.22	0.03	0.18
Braking force rate	F_1,15_ = 6.87	0.02	0.31	F_3,45_ = 0.30	0.82	0.02	F_3,45_ = 0.49	0.69	0.03
Peak propulsive force	F_1,15_ = 29.91	< 0.001	0.67	F_3,45_ = 0.66	0.58	0.04	F_3,45_ = 1.81	0.18	0.11
Propulsive force rate	F_1,15_ = 8.19	0.01	0.35	F_3,45_ = 2.21	0.13	0.13	F_3,45_ = 0.3	0.83	0.02
Mechanical efficiency	F_1,15_ = 8.66	0.01	0.37	F_3,45_ = 32.53	< 0.0001	0.68	F_3,45_ = 0.95	0.42	0.06
GL preactivation	F_1,13_ = 0.78	0.39	0.06	F_3,39_ = 0.21	0.89	0.02	F_1.37,17.83_ = 0.47	0.56	0.04
TA preactivation	F_1,13_ = 0.002	0.97	0.00	F_3,39_ = 0.67	0.58	0.05	F_3,39_ = 0.91	0.40	0.07
TA/GL preactivation	F_1,13_ = 0.79	0.39	0.57	F_3,39_ = 0.89	0.45	0.06	F_3,39_ = 0.45	0.72	0.03
TA-GL Coactivation	F_1,13_ = 1.02	0.33	0.08	F_3,39_ = 1.01	0.40	0.08	F_3,39_ = 0.60	0.62	0.05
RPE	F_1,15_ = 0.097	0.76	0.01	F_3,45_ = 85.47	< 0.0001	0.85	F_3,45_ = 0.11	0.88	0.01
Postexercise VAS	F_1,14_ = 44.95	< 0.0001	0.76	F_1.68, 23.48_ = 27.4	< 0.0001	0.66	F_2,28_ = 12.03	< 0.001	0.46

MinRS, minimal running shoes; TrdRS, traditional cushioned running shoes; C_r_, net energy cost of running; RER, respiratory exchange ratio; F_v,max_, maximal vertical ground reaction force; BM, body mass; TA, tibialis anterior; GL, gastrocnemius lateralis; RPE, rating of perceived effort; VAS, visual analog scale.

### Energetics of running

#### Net energy cost of running

**Figure 1:**
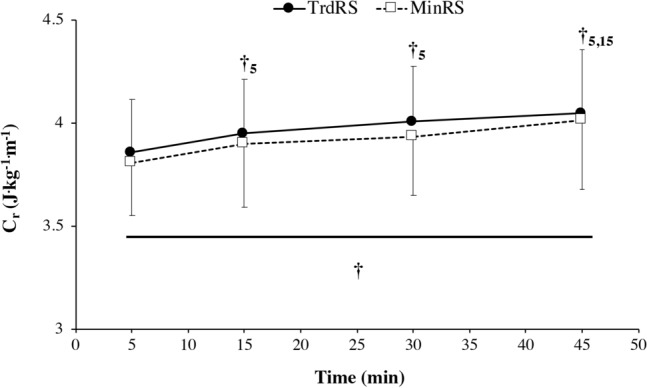
Net energy cost (C_r_) vs. time during the two experimental conditions [traditional cushioned (TrdRS) and minimalist (MinRS) running shoes]. Values are mean ± SD (n = 16). † indicates a significant time effect (*P* ≤ 0.05); †_5_ represents a significant difference from 5 min; †_15_ notes a significant difference from 15 min.

Participants ran at 11.8 ± 0.6 km/h. There was no significant time × condition interaction in C_r_ (*P* = 0.81). C_r_ was similar for both conditions (*P* = 0.25) with a significant increase over time (*P* < 0.0001; Fig. 1). C_r_ significantly increased at 15 and 30 min compared to 5 min (*P* < 0.0001 and *P* < 0.0001, respectively) and at 45 compared to 5 and 15 (*P* < 0.0001, *P* < 0.001, respectively).

#### Ventilation

**Figure 2:**
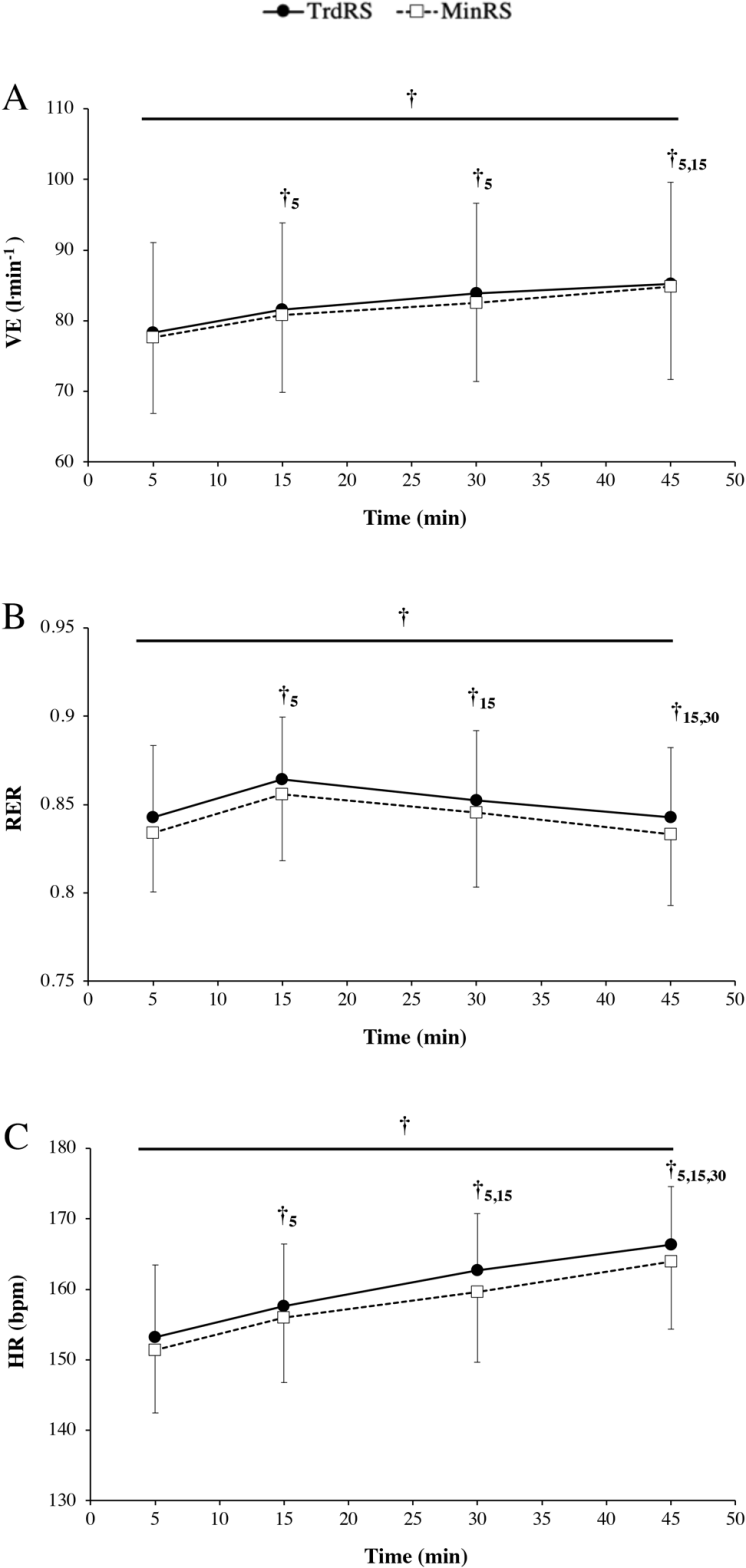
Ventilation (**A**), respiratory exchange ratio (RER) (**B**) and heart rate (**C**) versus time during the two experimental conditions [traditional cushioned (TrdRS) and minimalist (MinRS) running shoes]. Values are mean ± SD (n = 16). † indicates a significant time effect (*P* ≤ 0.05); †_5_ denotes a significant difference from 5 min; †_15_ represents a significant difference from 15 min; †_30_ indicates a significant difference from 30 min.

There was no significant time × condition interaction in the ventilation (*P* = 0.85). This variable was similar in both conditions (*P* = 0.56) and increased significantly over time (*P* < 0.0001; Fig. 2A). Post hoc analysis revealed a significant increase in ventilation at 15 and 30 min than at 5 min (*P* = 0.001 and *P* = 0.001, respectively) and at 45 min compared to 5 and 15 min (*P* < 0.0001 and *P* = 0.001, respectively).

#### Respiratory gas exchange

No significant time × condition interaction (*P* = 0.97) and difference in RER between conditions (*P* = 0.44) were noted. However, RER significantly changed over time (*P* < 0.0001; Fig. 2B). RER significantly increased at 15 compared to 5 and 30 min (*P* < 0.0001 and *P* < 0.0001, respectively) and significantly decreased at 45 min compared to 15 and 30 min (*P* < 0.0001 and *P* < 0.001, respectively).

#### Heart rate

There were no significant time × condition interaction (*P* = 0.47) and shoe condition (*P* = 0.13) in the heart rate. However, the heart rate increased significantly over time (*P* < 0.0001; Fig. 2C). Post hoc analysis revealed a significant increase in heart rate at 15, 30, and 45 min than at 5 min (*P* < 0.0001 for all) and at 45 min compared to 15 and 30 min (*P* < 0.0001 for both) and at 30 min than at 15 min (*P* = 0.0001).

### Biomechanics of running

#### Spatiotemporal parameters

No significant interaction (*P* ≥ 0.20) and evolution over time (*P* ≥ 0.25) were found in t_c_, step length and frequency. The step length was significantly shorter (*P* = 0.001) and step frequency was significantly higher in MinRS than in TrdRS (*P* = 0.001; Table 2). There was no significant difference in t_c_ (*P* = 0.032; Table 2).

**Table 2:** Spatiotemporal parameters, maximal vertical, braking and propulsive forces and loading, braking and propulsive rates and rating of perceived effort.

Variables	5 min		15 min		30 min		45 min	
	MinRS	TrdRS	MinRS	TrdRS	MinRS	TrdRS	MinRS	TrdRS
Step length (m)*	1.18 ± 0.08	1.21 ± 0.08	1.19 ± 0.08	1.21 ± 0.08	1.18 ± 0.08	1.21 ± 0.08	1.19 ± 0.08	1.23 ± 0.08
Step frequency (Hz)*	2.79 ± 0.13	2.73 ± 0.11	2.79 ± 0.13	2.72 ± 0.10	2.79 ± 0.13	2.72 ± 0.10	2.79 ± 0.13	2.69 ± 0.10
Contact time (ms)	251 ± 25	250 ± 11	245 ± 11	251 ± 12	246 ± 10	251 ± 12	245 ± 10	249 ± 13
Push time (ms)	130 ± 19	125 ± 5	126 ± 6	126 ± 6	126 ± 6	126 ± 6	126 ± 6	125 ± 7.8
Brake time (ms)*	121 ± 8	125 ± 7	120 ± 7	125 ± 7	120 ± 6	125 ± 8	119 ± 6	124 ± 8
Push time/brake time*	1.07 ± 0.11	1.01 ± 0.05	1.05 ± 0.05	1.01 ± 0.05	1.06 ± 0.06	1.01 ± 0.07	1.06 ± 0.06	1.01 ± 0.06
Lower limb deformation (cm)	15.1 ± 2.4	15.1 ± 1.0	14.6 ± 1.2	15.2 ± 1.0	14.6 ± 1.0	15.3 ± 1.3	14.5 ± 1.0	15.3 ± 1.3
F_v_,_max_ (BW)	2.52 ± 0.16	2.55 ± 0.19	2.53 ± 0.17	2.55 ± 0.17	2.51 ± 0.17	2.54 ± 0.15	2.50 ± 0.18	2.56 ± 0.16
Peak braking force (BW)	− 0.30 ± 0.04	− 0.29 ± 0.04	− 0.30 ± 0.03	− 0.29 ± 0.04	− 0.30 ± 0.03	− 0.30 ± 0.04	− 0.29 ± 0.04	− 0.30 ± 0.04
Peak propulsive force (BW)*	0.29 ± 0.02	0.27 ± 0.03	0.29 ± 0.03	0.27 ± 0.03	0.29 ± 0.02	0.27 ± 0.03	0.29 ± 0.02	0.27 ± 0.03
Loading rate (BW s^−1^*)*	53.5 ± 13.2	56.7 ± 7.9	57.2 ± 13.2	57.7 ± 7.2	57.3 ± 13.5	57.3 ± 7.6	57.9 ± 14.1	57.9 ± 8.0
Braking force rate (BW s^−1^)	− 4.0 ± 1.6	− 4.6 ± 1.6	− 3.8 ± 1.7	− 4.6 ± 1.6	− 3.9 ± 1.8	− 4.6 ± 1.7	− 3.8 ± 1.8	− 4.7 ± 1.4
Propulsive force rate (BW s^−1^)	5.6 ± 0.7	5.3 ± 1.0	5.6 ± 0.8	5.3 ± 0.9	5.5 ± 0.8	5.2 ± 0.9	5.5 ± 0.8	5.2 ± 1.0
Rating of perceived effort	10.7 ± 1.6	10.5 ± 1.6	11.4 ± 1.3	11.4 ± 1.3	12.8 ± 1.7	12.6 ± 1.6	13.1 ± 1.2	13.2 ± 1.6

Values are mean ± SD (n = 16).

F_v,max_, maximal vertical force; BW, body weight.

* indicates a significant difference between conditions (*P* ≤ 0.05).

#### Mechanical works

**Figure 3:**
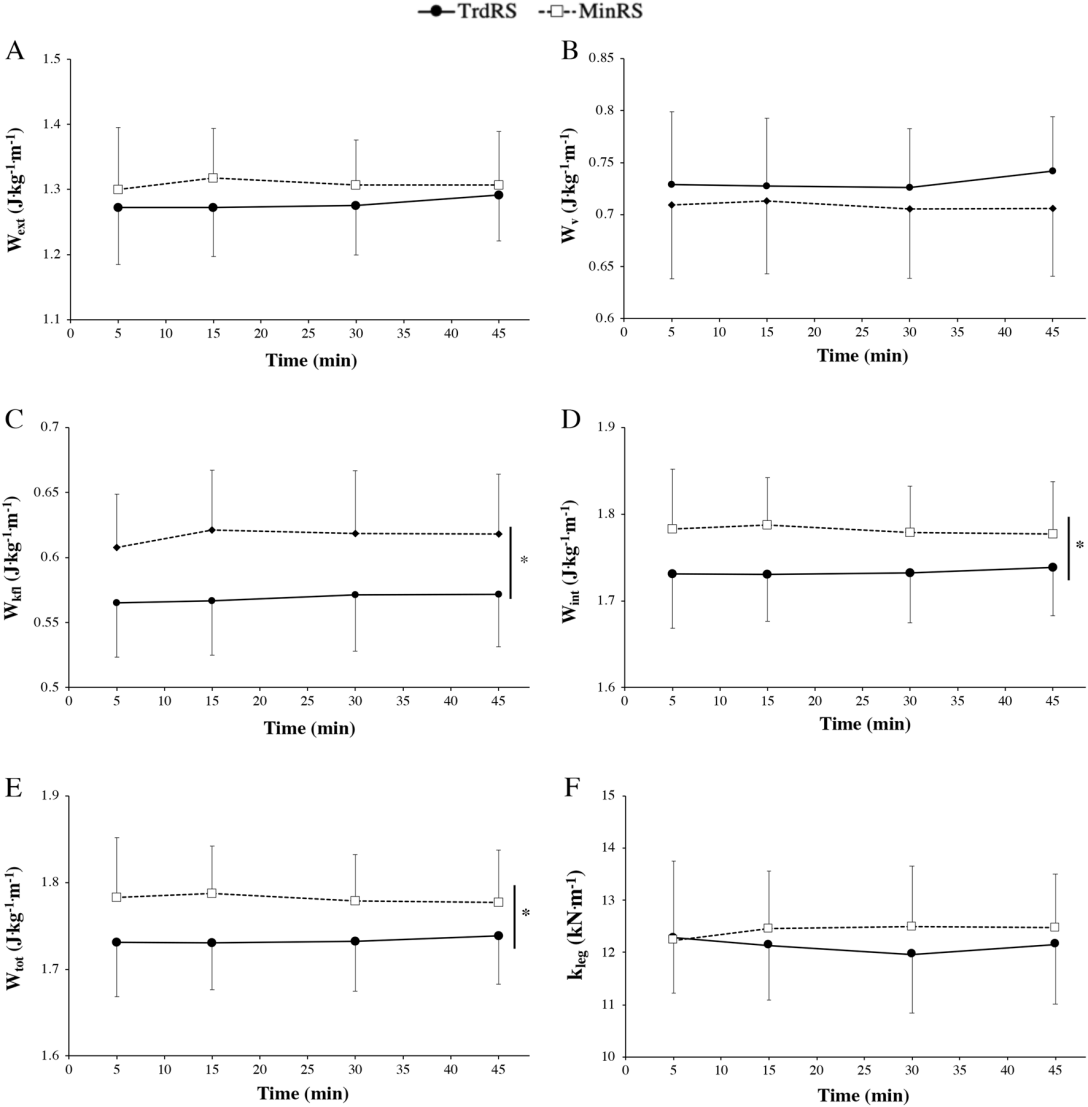
External mechanical work (W_ext_) (**A**), vertical mechanical work (W_v_) (**B**), kinetic fore-aft and lateral mechanical work (W_kfl_) (**C**), internal mechanical work (W_int_) (**D**), total mechanical work (W_tot_) (**E**) and leg stiffness (k_leg_) (**F**) versus time during the two experimental conditions [traditional cushioned (TrdRS) and minimalist (MinRS) running shoes]. Values are mean ± SD (n = 16). * indicates a significant condition effect (*P* ≤ 0.05).

There were no significant time × condition interaction, shoe condition and evolution over time in the external (*P* = 0.27, *P* = 0.02 and *P* = 0.35, respectively) and vertical (*P* = 0.29, *P* = 0.01 and *P* = 0.45, respectively) mechanical works per unit distance (Fig. 3 A,B). No significant time × condition interaction (*P* = 0.42) and evolution over time (*P* = 0.22) were found fore-aft and lateral work per distance. However, this latter was significantly higher in MinRS compared with TrdRS (*P* < 0.0001; Fig. 3C).

There were no significant time × condition interaction (*P* = 0.26) and evolution over time (*P* = 0.85) in the internal mechanical work per unit distance which was significantly higher in MinRS compared with TrdRS (*P* < 0.001, Fig. 3E).

No significant time × condition interaction (*P* = 0.26) and evolution over time (*P* = 0.85) were noted in the total mechanical work. However, this work was significantly higher in MinRS compared with TrdRS (*P* = 0.001; Fig. 3D).

#### Leg stiffness

No significant variation in the time × condition interaction, shoe and time conditions were found in F_v,max_/BM (*P* = 0.44, *P* = 0.04 and *P* = 0.56, respectively; Table 2), lower limb deformation (*P* = 0.18, *P* = 0.02 and *P* = 0.57, respectively; Table 2) and k_leg_ (*P* = 0.11, *P* = 0.05 and *P* = 0.92, respectively; Fig. 3F).

#### Braking and propulsive durations and forces and loading, braking and propulsive rates

No significant interaction (*P* = 0.33), time (*P* = 0.33) and condition (*P* = 0.21) effects were noted in the push time (Table 2). There were no significant time × condition interaction (*P* = 0.43) and time (*P* = 0.29) effects in the brake time with this variable which was significantly shorter in MinRS than in TrdRS (*P* = 0.001; Table 2). These findings induced a significantly higher push-to-brake time ratio in the former compared with the latter (*P* = 0.001) with no significant time or interaction effects (*P* = 0.69 and *P* = 0.59, respectively; Table 2).

No significant interaction (*P* = 0.34), time (*P* = 0.56) and condition (*P* = 0.63) effects were noted in the loading rate (Table 2).

There was no significant time × condition interaction (*P* = 0.03) in the peak braking force with no significant evolution over time (*P* = 0.63) and difference between the two conditions (*P* = 0.68; Table 2). No significant interaction (*P* = 0.56), shoe (*P* = 0.02) and time (*P* = 0.76) effects were found in the braking rate. There were no significant time × condition interaction (*P* = 0.18) and change over time (*P* = 0.58) in the peak propulsive force which was significantly higher in MinRS compared with TrdRS (*P* < 0.0001; Table 2). No significant interaction (*P* = 0.83), shoe (*P* = 0.01) and time (*P* = 0.13) effects were found in the propulsive force rate (Table 2).

### Mechanical efficiency

**Figure 4:**
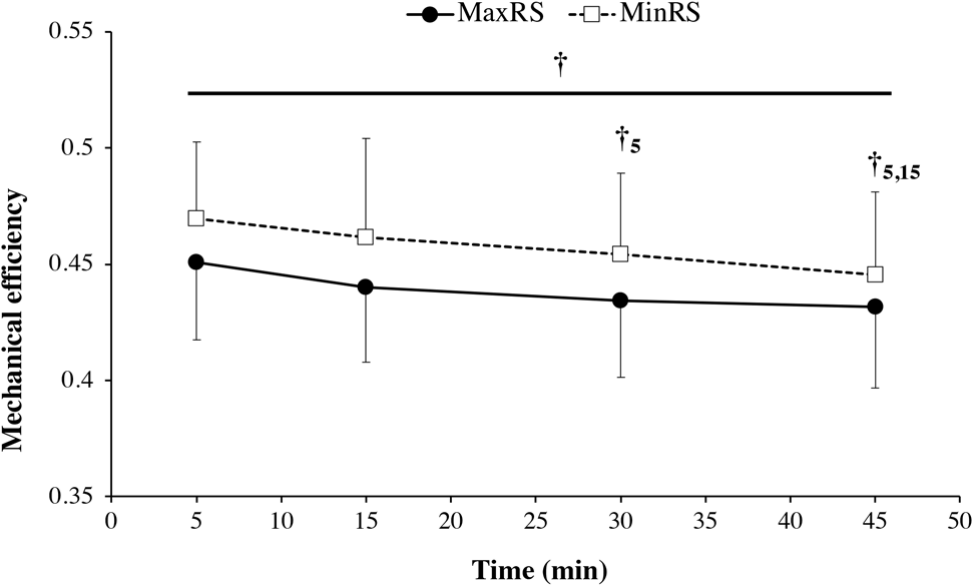
Mechanical efficiency versus time during the two experimental conditions [traditional cushioned (TrdRS) and minimalist (MinRS) running shoes]. Values are mean ± SD (n = 16). † denotes a significant time effect (*P* ≤ 0.05); †_5_ indicates a significant difference from 5 min; †_15_ represents a significant difference from 15 min.

No significant time × condition interaction (*P* = 0.42) and shoe condition (*P* = 0.01) were noted in mechanical efficiency. However, this variable significantly decrease over time (*P* < 0.0001; Fig. 4).

Pairwise comparison revealed a significant decrease in mechanical efficiency at 30 than at 5 (*P* < 0.001) and at 45 min compared to 5 and 15 min (*P* < 0.001 for both; Fig. 4).

### Muscle preactivation and muscle coactivation during the contact phase

**Figure 5:**
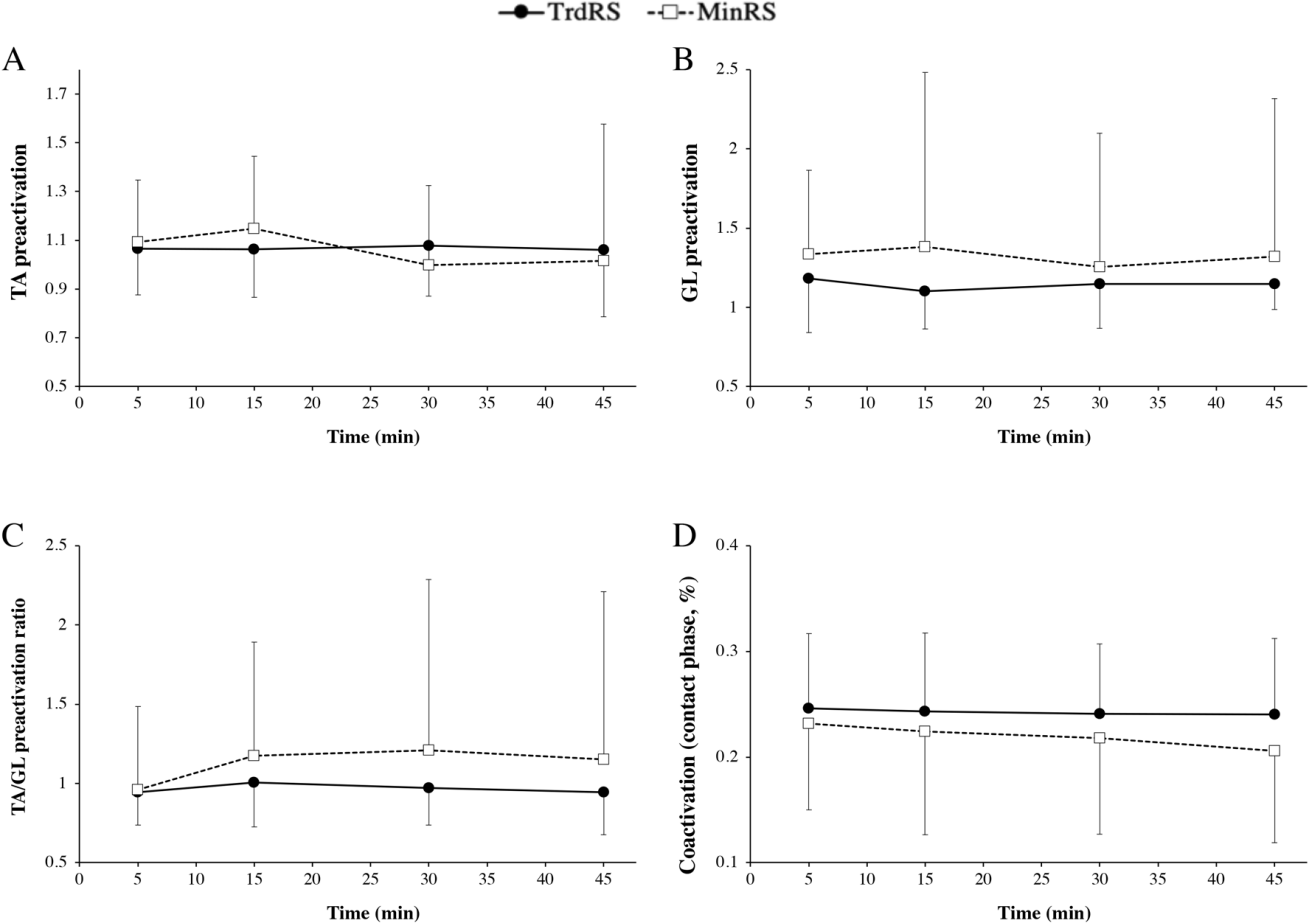
Tibialis anterior (TA) (**A**) and gastrocnemius lateralis (GL) (**B**) preactivation, TA/GL preactivation ratio (**C**) and TA and GL coactivation during the contact phase (**D**) versus time during the two experimental conditions [traditional cushioned (TrdRS) and minimalist (MinRS) running shoes]. Values are mean ± SD (n = 14).

TA and GL preactivation, TA/GL preactivation ratio and TA and GL coactivation during the contact phase showed no significant time × condition interaction (*P* ≥ 0.40), time (*P* ≥ 0.40) and condition (*P* ≥ 0.33) effects (Fig. 5).

### Rating of perceived effort and postexercise lower limb muscle pain

There were no significant time × condition interaction (*P* = 0.88) and condition (*P* = 0.76) effects in the rating of perceived effort. However, this variable increased significantly over time (*P* < 0.0001; Table 2). A significant time × condition interaction was found in the postexercise visual analog scale (*P* < 0.0001). This latter was significantly higher in MinRS compared with TrdRS (24 h: 5.0 ± 2.0 and 2.1 ± 1.2, 48 h: 4.1 ± 1.83 and 1.6 ± 0.7, and 72 h: 2.1 ± 1.0 and 1.0 ± 0.0, in MinRS and TrdRS, respectively; *P* < 0.0001) and significantly decreased at 72 h compared to 24 and 48 h (*P* < 0.0001 for both) with no significant difference between 24 and 48 h (*P* = 0.08) in MinRS. In contrast, no significant evolution over time was found in TrdRS (*P* ≥ 0.003).

## Discussion

Our findings showed that at the beginning of the trial (5 min), C_r_ and muscle pre- and co-activation were not significantly different between minimalist and traditional cushioned running shoes, and step frequency and k_leg_ (although nonsignificant for this latter) were higher in the former compared with the latter. This finding partially confirms the first hypothesis of this study. Moreover, C_r_ significantly increased during the 45-min trial but with no significant difference between shoe wear conditions along with no significant change over time in step frequency, k_leg_, total mechanical work and muscle pre- and co-activation for both conditions in contrast with the second hypothesis of this study. Only postexercise muscle pain was higher in minimalist shoes compared with traditional cushioned shoes.

To the best of our knowledge, this study is the first to investigate the time course of C_r_ in MinRS versus TrdRS during moderate-intensity exercise. During the whole exercise duration, our results showed that C_r_ was not significantly different between the shoe conditions with both increasing over time (~ + 5% between 5 and 45 min; Fig. 1). This increased C_r_ was associated with no significant change in muscle activations and total mechanical work over time induced a significant decrease in mechanical efficiency (~ − 5%) with exercise duration, which may be detrimental for endurance running performance. Therefore, this alteration in running economy was not related to neuromechanical changes in the running pattern but due to physiological modifications with exercise duration. This upward drift in C_r_ during moderate and prolonged (> 30 min of duration) exercise may thus result from increasing blood levels of catecholamines, metabolic cost of ventilation, body temperature, and shifting in substrate utilization due to increased lipid oxidation over time^35,36^. This finding was partially corroborated by our results showing a significant increase in heart rate and ventilation between 5 and 45 min (~ + 8% and + 9%, respectively) with no significant change in RER between these time points (Fig. 2).

These results are not completely consistent with previous studies investigating the effect of duration on the energetics and biomechanics of running in MinRS and TrdRS^22,23^. Vercruyssen et al.^23^ found that after a short-distance trail, C_r_ was increased only in MinRS with a more pronounced decrease in k_leg_ and increase in footstrike angle compared with TrdRS. Moreover, the post-trail muscle calf pain increased significantly during level running only in MinRS compared with TrdRS. The latter result is indirectly corroborated by our higher post-exercise lower limb muscle pain in minimalist vs. traditional cushioned shoes even if our participants were familiarized with MinRS with 2 weeks (4 training sessions) of gradual exposition to this type of shoe. This increased post-exercise muscle pain should have been associated with increased muscle activation in MinRs vs. TrdRS and a more pronounced increase over time in this variable in the former than in the latter, as previously suggested^7,21^. However, our results of muscle pre- and co-activation during the contact phase did not present these differences between the two shoe conditions and over time (Fig. 5). This finding could be due to the well-known limitation in using dynamic EMG during running to assess muscle activation^37^, which could be even more important with our measurements during the 45-min submaximal exercise. The high variability in our EMG data indicates that the use of this parameter is hence limited and has to be interpreted with caution.

Another study^22^ investigating the biomechanical changes during 50 min of mixed-profile treadmill running [5 blocks of 5 min of flat, ascent (5%) and descent (-5%)] reported that flight time, plantar flexion, foot angles, and k_leg_ significantly decreased over time only in MinRS and not in TrdRS even if the difference between the 2 shoe conditions disappeared after 30 min of running. This finding is in contrast with our findings showing no significant changes in spatiotemporal parameters (Table 2) and k_leg_ (Fig. 3F) over time in either shoe condition. However, our results corroborated those reporting that k_leg_ did not change after exercise and was similar in running modality (level running), duration (60 min), and intensity (moderate intensity) to that used in the present study but performed in traditional cushioned shoes^38^. Therefore, longer exhaustive or more intensive exercise seems to be required to induce neuromuscular fatigue and decrease k_leg_^22^. Nevertheless, the difference between our findings and those of previous studies may also be due to the differences in fatiguing exercises used (level vs. trail or mixed profile running). Moreover, compared with previous studies, the originality of our results was to simultaneously assess the time course of change in energetics and biomechanics of running as previously suggested^23^.

Our results showed no significant difference between the 2 shoe conditions in contrast to the recent meta-analytical review reporting a C_r_ slightly lower in MinRS than in TrdRS^6^. However, the findings of the present study confirmed the typical biomechanical differences between these conditions, such a higher step frequency (Table 2) and k_leg_ (although nonsignificant for this latter; Fig. 3F) in MinRS compared with TrdRS^22,39,40^, but extended these results over time (45 min of level running at moderate intensity) and added the assessment of mechanical works during this exercise. Our findings showed that total and internal mechanical works were higher in MinRS compared with TrdRS (~ + 2.7%, + 4.8%, respectively; Fig. 3). According to the equation used to estimate the internal mechanical work^27^, the higher internal mechanical work was due to the higher step frequency in MinRS vs. TrdRS. The higher but nonsignificant external mechanical work in the former compared with the latter was due to the greater fore-aft and lateral work (+ 8%), which was associated with lower vertical mechanical work (− 4.5%). A greater variation in v_h_ is involved in this greater fore-aft and lateral work in MinRS. This finding was essentially due to a higher average braking force during the contact phase in MinRS than in TrdRS. In fact, a more landing-takeoff asymmetry in the former (i.e., higher push time/brake time; Table 2) implies a higher average breaking force than the average propulsive force in this shoe condition^41^. However, this extra-fore-aft and lateral work did not require an increase in C_r_ because this greater braking force would allow the muscle–tendon units to store and then release a low-metabolic cost elastic energy, as indirectly attested by nonsignificant higher values of k_leg_ (+ 2.3%) and mechanical efficiency (+ 4.2%) in MinRS than in TrdRS (Figs. 3F and 4). These results partially confirm those of Ardigo et al.^42^ showing a higher forward kinetic work but similar vertical work in forefoot running compared with rear foot running, inducing higher external and total mechanical works and similar C_r_ in the former compared with the latter.

On the other hand, the higher internal mechanical work, step frequency and average breaking force in MinRS compared with TrdRS may penalize C_r_ in minimalist shoes. In fact, the optimal step frequency (i.e., step frequency that minimizes C_r_) is influenced by the tradeoff between the cost of swinging the lower limbs and the cost of braking forces related to the anteroposterior foot position relative to the hip (i.e., ~ center of body mass position) at landing^43^. This feature may negatively compensate for the energetic advantage due to the better use of the elastic energy in MinRS and contribute to increasing Cr under this shoe condition, making it similar between MinRS and MaxRS.

Another supposed advantage for minimalist shoes is the reduced loading rates^1^ associated with lower injury risks^44^ even if it has been recently shown that there is inconsistent^45^ or no^46^ evidence for the association between loading rate and injury risks. In our study, there was no significant difference in loading rate between MinRS and TrdRS (Table 2) in contrast with previous findings^1^. However, our results are consistent with those of Tam et al.^47^, revealing a similar loading rate between experienced traditional cushioned and minimalist runners. The former may use the additional cushioning provided by their traditional cushioned shoes to attenuate the greater loading rate typically found in these runners compared with minimalist runners^47^. Moreover, this result still indirectly attests that our runners are properly familiarized with MinRS. The gradual exposition to minimalistic shoes during the 2-wk familiarization protocol allows our runners to obtain a nonsignificant lower braking force rate in MinRS compared with TrdRS thanks to a longer duration between initial ground contact and peak braking force in the former compared with latter conditions. In MinRS, our runners adopted this “protective” strategy to decrease the impact of the higher average braking force during the initial contact phase as previously also reported by others^48^.

This study has some methodological limitations. First, we tested only one endurance running speed (11.8 ± 0.6 km/h), and these results should be confirmed with faster running speeds. However, we used this speed, corresponding to 95% of the ventilatory threshold, to accurately assess C_r_ with indirect calorimetry. Second, a quantitative assessment of foot strike patterns^49^ in the two shoe conditions could have been complementary to the measurements performed in this study. Nevertheless, the experimenters visually and quantitatively determined the foot strike pattern of the runners watching 3 video tracks of 30 s acquired at 5, 15 and 40 min during the two shoe condition trials for each runner. Comparing TrdRS to MinRS, 11 runners changed the foot strike pattern from the rear- to midfoot strike pattern, 1 changed from the mid- to forefoot strike pattern, and 4 runners did not change their rearfoot strike pattern. In MinRS, only 2 runners changed the foot strike pattern one time (from 5 to 15 min) during the 45-min running bout: one changed from the rear- to midfoot and one from the fore- to midfoot strike pattern. In TrdRS, only one runner changed between 5 and 15 min from the rear to midfoot. Future studies should thus include a quantitative assessment of foot strike patterns^49^ to isolate the effect of these patterns and the type of shoes on the energetics and mechanics of running and their changes over time.

## Conclusions

In conclusion, during 45-min submaximal running, our findings showed that C_r_ and muscle pre- and co-activation were not significantly different between minimalist and traditional cushioned running shoes with significantly higher step frequency and total mechanical work, and nonsignificant greater k_leg_ noted in the former than in the latter. The similar C_r_ between the two shoe conditions may be explained by the better use of the elastic energy in MinRS that is subsequently penalized by the metabolic cost associated with the higher step frequency and average braking force compared with TrdRS. Moreover, C_r_ significantly increased during the 45-min trial in both shoe conditions along with no significant change over time in muscle activation and biomechanical variables. Therefore, this alteration in the running economy may not be related to the neuromechanical changes in the running pattern but likely due to physiological modifications with exercise duration.

## Data Availability

The datasets used and/or analyzed during the current study are available from the corresponding author on reasonable request.

## Abbreviations

ΔL: Lower limb deformation
C_r_: Net energy cost of running
DF: Duty factor
E_kfl_: Fore-aft and lateral kinetic energies of the center of mass
E_kv_: Vertical kinetic energy of the center of mass
EMG: Surface electromyography
E_p_: Potential energy of the center of mass
E_v_: Vertical energy of the center of mass
F_v,max_: Maximal vertical ground reaction force
g: Acceleration of gravity
GL: Gastrocnemius lateralis
k_leg_: Leg stiffness
L: Initial leg length
m: Body mass
MinRS: Minimalist running shoes
TrdRS: Traditional cushioned running shoes
TA: Tibialis anterior
RER: Respiratory exchange ratio
SF: Stride frequency
t_c_: Contact time
t_f_: Fly time
v: Running speed
V_h_: Horizontal velocity of the center of body mass
V_l_: Lateral velocity of the center of body mass
V_v_: Vertical velocity of the center of body mass
